# Bone Marrow Niches for Hematopoietic Stem Cells and Immune Cells

**DOI:** 10.2174/187152812800392689

**Published:** 2012-06

**Authors:** Tatsuki Sugiyama, Takashi Nagasawa

**Affiliations:** 1 Department of Immunobiology and Hematology, Institute for Frontier Medical Sciences, Kyoto University, 53 Kawahara-cho, Shogoin, Sakyo-ku, Kyoto 606-8507, Japan; 2 Japan Science and Technology Agency (JST), Core Research for Evolutional Science and Technology (CREST), Tokyo 102-0075, Japan

**Keywords:** Bone marrow, chemokine, CXCL12, HSC, niche, SCF, stromal cell.

## Abstract

In mammals, hematopoietic stem cells (HSCs), which give rise to all blood cells and their progenies, including immune cells are controlled by special microenvironments, termed niches in the bone marrow during homeostasis and infection. However, the identity, nature and function of these niches remain unclear. It has been reported that HSCs are in contact with osteoblasts lining the bone surface and osteoblasts act as niches for HSCs (termed endosteal niche). However, recent studies suggest that only a small number of HSCs reside in the endosteal niche. In contrast, many HSCs are shown to be in contact with endothelial cells in the marrow. In addition, recent studies suggest that primitive mesenchymal cells, including CXCL12-abundant reticular (CAR) cells and Nestin-expressing cells, which have the ability to differentiate into adipocytes as well as osteoblasts act as niches for HSCs. Here we review candidate niches for HSCs in the bone marrow controlling hematopoiesis and chronic inflammation.

## INTRODUCTION

Hematopoietic stem cells (HSCs) give rise to all lineages of blood cells, including immune cells within the bone marrow. The bone marrow is surrounded by bone surface and occupies the medullary cavities of bones throughout the skeleton and contains a dense network of medullary vascular sinuses, and blood cells and their precursors are packed in the extravascular spaces between the sinuses. It has been assumed that HSCs and hematopoietic progenitors reside in the microenvironments in the bone marrow, known as niches, which provide HSCs and hematopoietic progenitors with regulatory signals essential for their maintenance, proliferation and differentiation to produce the appropriate numbers of mature blood cells throughout life [1-3]. However, niche identity and function are subjects of longstanding debate.

In 1977, a liquid culture system, in which primitive hematopoietic cells proliferated *in vitro* for several months, was reported. This suggests that adherent stromal cells in the culture, which might include osteoblasts, endothelial cells and macrophages, support hematopoiesis [4, 5]. On the other hand, ultrastructural studies suggest that morphologically termed adventitial reticular cells that surround vascular sinuses are the dominant stromal cells found in bone marrow [6]. However, adventitial reticular cells lack distinctive characteristics, making it difficult to prove their nature and functions as a niche for hematopoiesis *in vivo*.

## THE ENDOSTEAL NICHE: OSTEOBLASTS, SNO CELLS AND OSTEOCLASTS

In 1994, primary human osteoblasts were shown to maintain the proliferation of primitive hematopoietic progenitors *in vitro*, raising the possibility that osteoblasts are involved in hematopoiesis [7]. Osteoblasts line the bone surface and are involved in synthesis, deposition and mineralization of extracellular matrix of the bone and play a central role in bone development. Subsequently, it was reported that parathyroid hormone (PTH) treatment increases the numbers of osteoblasts in culture, and increases HSC numbers *in vivo* [8]. In addition, cells which retained BrdU after treatment for 10 days followed by a 70-day chase were identified as HSCs, and these cells were found in contact with a population of osteoblasts lining the bone surface, termed spindle-shaped N-cadherin^+^CD45^-^ osteoblastic (SNO) cells, which express a high level of N-cadherin [9]. SNO cells line the bone surface but are morphologically different from cuboidal (active) osteoblasts. The numbers of SNO cells are increased in bone morphogenetic protein (BMP) receptor type IA (BMPRIA) conditionally deficient mice and this correlates with an increase in the numbers of HSCs [9]. In addition, cells expressing Tie2^+^ and N-cadherin were identified as HSCs and found in contact with bone-lining osteoblasts expressing osteocalcin in mice myelosuppressed with 5-FU [10]. On the other hand, depleting cells of the osteoblastic lineage using a 2.3-kb fragment of the rat type I collagen α1 (Col1α1) gene promoter driving thymidine kinase expression (Col2.3∆TK), induced a marked reduction in B cells and erythroid progenitors [11, 12]. These results suggested that bone-lining osteoblasts, including SNO cells function as a niche for HSCs and hematopoietic progenitors (termed endosteal niches).

However, recent studies complicate the data presented above. It was reported that only 4.6% of HSCs retained BrdU after 10 days treatment and a 70-day chase [13], few CD150^+^CD48^-^CD41^-^Lin^-^ HSCs were adjacent to the bone surface in bone marrow sections [14], and that hematopoiesis appeared normal in N-cadherin conditionally-deficient mice [15]. Additionally, the receptor for PTH/PTH-related protein (PPR), BMPRIA and Col1α1 are expressed in candidate cellular niches other than osteoblasts, including CXC chemokine ligand (CXCL) 12-abundant reticular (CAR) cells, described below [16]. Furthermore, the numbers of HSCs and blood cells were not reduced in the mice, in which osteoblast numbers were reduced [2, 14] and HSC numbers were not affected when stem cell factor (SCF), which is essential for HSC maintenance, was conditionally deleted from osteoblasts [17]. Thus, it remains unclear if osteoblasts are a key component of HSC niches.

Osteoclasts, which are generated from HSCs and resorb the mineralized bone matrix formed by chondrocytes or osteoblasts, are located in endosteal niches. The role of osteoclasts in hematopoiesis remains a controversial issue. It has been reported that osteoclasts degrade endosteal niche components and enhance mobilization of hematopoietic progenitor cells [18]. In contrast, experiments using mice treated with zoledronate, which inhibit osteoclasts suggested that mobilization of hematopoietic progenitor cells in response to G-CSF is not mediated by osteoclasts [19]. Further studies will be needed to clarify how osteoclasts regulate hematopoietic stem and progenitor cell behavior.

## THE RETICULAR NICHE FOR HEMATOPOIESIS: CXCL12-ABUNDANT RETICULAR (CAR) CELLS

The studies focusing on the chemokine CXCL12 revealed that the specialized reticular cells in marrow cavities function as a niche for hematopoiesis. Chemokines are a large family of structurally related chemoattractive cytokines, which act through seven-transmembrane-spanning receptors coupled to heterotrimeric GTP-binding proteins (G-protein-coupled receptors) [20]. CXCL12 (also known as stromal cell-derived factor [SDF]-1), for which the physiological receptor is CXC-chemokine receptor 4 (CXCR4), was first characterized as a growth stimulating factor for a B-cell precursor clone [21-25]. Gene targeting studies have revealed that CXCL12-CXCR4 signaling is essential for homing and maintenance of HSCs and development of immune cells, including B cells, plasmacytoid dendritic cells (pDCs), and NK cells in bone marrow [22-30].

**Fig. (1):**
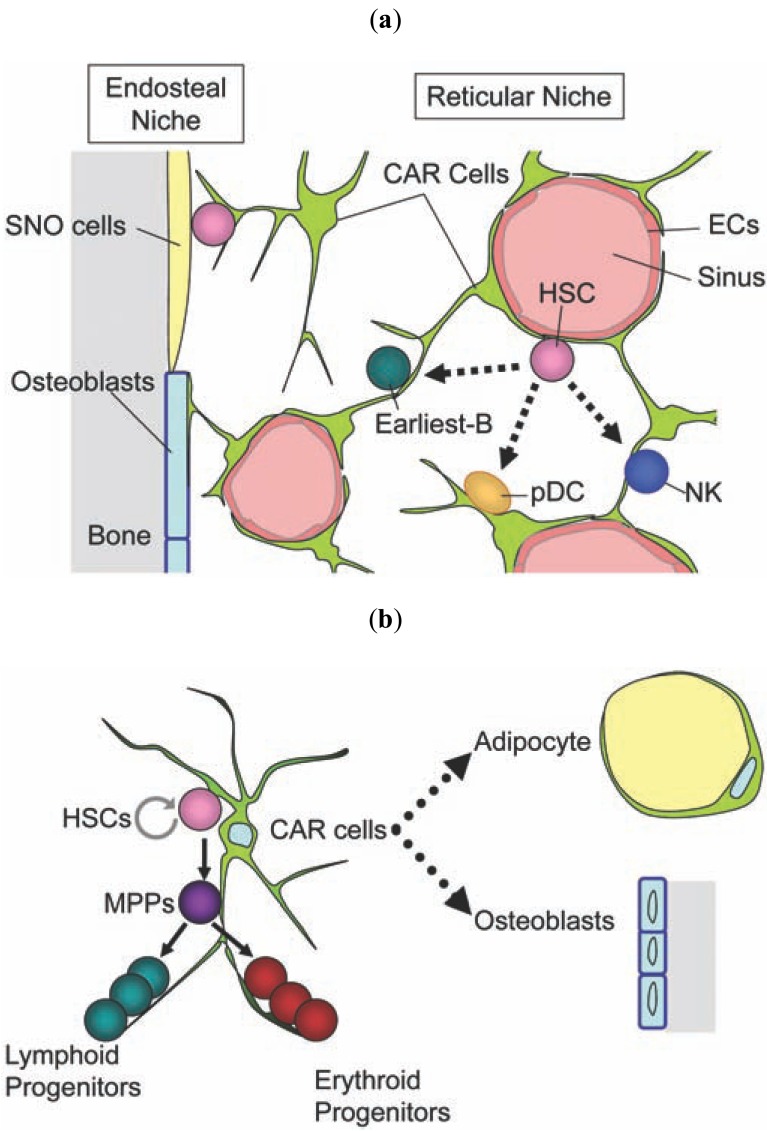
Mesenchymal progenitors might be key components of niches that maintain and regulate HSCs and hematopoietic progenitors. (**a**) A model for the localization of HSCs and their association with candidate cellular niches in the bone marrow. Most HSCs are in contact with the processes of CAR cells, which are the major producer of essential hematopoietic cytokines, including CXCL12 and SCF. CAR cells act as niches for HSCs and hematopoietic progenitors. Many HSCs are in contact with CAR cells surrounding sinusoidal endothelial cells. In contrast, a relatively small population of HSCs are in contact with bone-lining osteoblasts, including SNO cells, which express a high level of N-cadherin. It has been reported that the cells which express Nestin in the bone marrow are mesenchymal stem cells (MSCs) and function as a niche for HSCs and that Sca-1^+^ PDGFRα^+^ subendothelial cells are MSCs in the marrow. However, the relationship of the Nestin-expressing cells with CAR cells and Sca-1^+^ MSCs remains unclear. (**b**) CAR cells are mesenchymal progenitors, which have potential to differentiate into adipocytes and osteoblasts, and esseential for proliferation of lymphoid and erythroid progenitors and HSCs, and maintenance of HSCs in an undifferentiated state. MPP; multipotent progenitors.

Immunohistochemical analysis of mice containing the green fluorescent protein (GFP) gene knocked into the CXCL12 locus (CXCL12-GFP mice) revealed that high CXCL12-GFP expression was detected in a small population of reticular cells, termed CXCL12-abundant reticular (CAR) cells, which are uniformly scattered throughout the bone marrow and have the long processes creating a network [29, 30] (Fig. **1a**). In addition, most CD150^+^CD48^-^CD41^-^ HSCs (97%), earliest B cell precursors, plasma cells, pDCs and NK cells were in contact with the processes of CAR cells in the marrow, suggesting that CAR cells function as a niche for HSCs and all immune cells produced in bone marrow (reticular niches) [26, 27, 29, 30] (Fig. **1**).

Histochemical analysis revealed that all bone marrow sinusoidal endothelial cells are surrounded by a proportion of CAR cells [30] (Fig. **1a**); however, CAR cells do not express the pan-endothelial marker platelet/endothelial cell-adhesion molecule 1 (PECAM-1)/CD31 or the smooth muscle cell maker and smooth muscle α-actin (SMαA), suggesting that they are different from endothelial cells and smooth muscle cells [29]. Flow cytometric analysis revealed that CAR cells do not express CD45, Sca-1 or CD31 and exhibit a mostly homogeneous expression of VCAM-1, CD44, platelet-derived growth factor receptor (PDGFR)α and PDGFRβ, suggesting that CAR cells consist of a relatively homogeneous population and can be largely identified as CD45^-^CD31^-^Sca-1^-^PDGFRβ^+^ cells [16].

To clarify the *in vivo* role of CAR cells, a diphtheria toxin receptor (DTR) mouse model selectively ablating CAR cells has been generated, in which the numbers of CAR cells are severely reduced but other candidate niches, including bone-lining osteoblasts or endothelial cells are not affected 2 days after DT administration [16]. HSCs from the CAR cell-depleted mice were reduced in number (about 2-fold) and cell size, were more quiescent and had increased expression of genes involved in myeloid fate decision and differentiation. Furthermore, the numbers of cycling B cell and erythroid progenitors were reduced in the CAR cell-depleted mice. In CXCL12-GFP mice, the expression of CXCL12 and SCF, which is essential for the proliferation of B cell and erythroid progenitors as well as HSCs was higher in sorted CAR cells than in other populations from bone marrow and bone fractions, and individual CAR cells express both CXCL12 and SCF. Consistent with these, the short-term ablation of CAR cells *in vivo* impaired SCF and CXCL12 production, indicating that CAR cells are the major producer of CXCL12 and SCF in the bone marrow [16]. It has been shown that the majority of individual CAR cells express both adipogenic and osteogenic genes, including PPARγ, runx2 and osterix (Osx) and have the potential to differentiate into adipocytes and osteoblasts in culture. Consistent with this, short-term ablation of CAR cells *in vivo* impaired the adipogenic and osteogenic differentiation potential of bone marrow cells [16]. These results suggest that CAR cells are adipo-osteogenic progenitors that are required for the proliferation of B cell and erythroid progenitor and HSCs, and for maintaining HSCs in an undifferentiated state. Processes of CAR cells might provide hematopoietic stem and progenitor cells with key regulatory cytokines [16] (Fig. **1a**) although it remains unclear what determines the specificity of interaction between certain hematopoietic cells and CAR cells.

In human bone marrow, the CD146-expressing, subendothelial cells are shown to be skeletal progenitors [31]. The abundant CXCL12 expression, multipotency, cell morphology and location of CD146-expressing subendothelial cells suggest that these cells are the human counterparts of CAR cells.

## NESTIN-EXPRESSING CELLS

Nestin is an intermediate filament protein that was originally identified as a marker of neural progenitors [32]. Its expression has subsequently been detected in a wide range of progenitor cells and endothelial cells [33]. It was recently reported that in bone marrow from transgenic mice in which GFP is expressed under the control of the neural-specific regulatory elements of the *Nes *(Nestin) gene, GFP expressing cells, termed Nes-GFP^+^ cells showed an exclusive perivascular distribution, and that many CD150^+^CD48^-^Lin^-^ HSCs (60%) were adjacent to Nes-GFP^+^ cells. In addition, mRNA expression of CXCL12 and SCF was much higher in sorted CD45^-^Nes-GFP^+^ cells, compared with sorted CD45^-^Nes-GFP^-^ non-hematopoietic cells. Furthermore, HSC numbers were reduced about 2-fold in the marrow but increased in the spleen 16 days after *in vivo* depletion of nestin-Cre-expressing cells using a DTR-mediated cell knockout technique, suggesting that nestin-expressing cells are involved in retaining HSCs in the bone marrow.

Sorted Nes-GFP^+^ cells contained cells that formed mesenchymal spheres and differentiate into adipocytes and osteoblasts, and colony-forming units-fibroblasts (CFU-F). Based on these findings, it was concluded that Nes-GFP^+^ cells are mesenchymal stem cells [34] (Fig. **1**). However, the frequencies of Nes-GFP^+^ cells with ability to give rise to multiple cell types is unclear, and only about 6.9% or 0.7% of Nes-GFP^+^ cells possess the ability to form mesenchymal spheres or CFU-F, respectively [34], raising the possibility that many Nes-GFP^+^ cells are not mesenchymal stem cells. In addition, a recent study has shown that nestin-expressing cells in the marrow contain some neural cells [35]. Abundant expression of CXCL12 and SCF in sorted Nes-GFP^+^ cells suggests that nestin-expressing cells contain CAR cells [34]; however, significant mRNA expression of nestin was not detected in SCF-expressing cells, which would largely equate to CAR cells [17]. Consistent with this, HSCs were not affected when SCF was conditionally deleted from nestin-Cre-expressing cells [17]. On the other hand, Sca-1^+^CD45^-^Ter119^-^PDGFRα^+^CD34^+^ cells, which had much lower levels of CXCL12 mRNA expression than Sca-1^-^PDGFRα^+ ^non-hematopoietic cells (including CAR cells) have been reported as a candidate for bone marrow mesenchymal stem cell population [36]. Thus, it will be important to address the relationship of nestin-expressing cells with the Sca-1^+^ mesenchymal stem cell population as well as CAR cells and neural cells in the marrow.

## THE ROLE OF CAR AND NESTIN-EXPRESSING CELLS IN CHRONIC INFLAMMATION

**Fig. (2):**
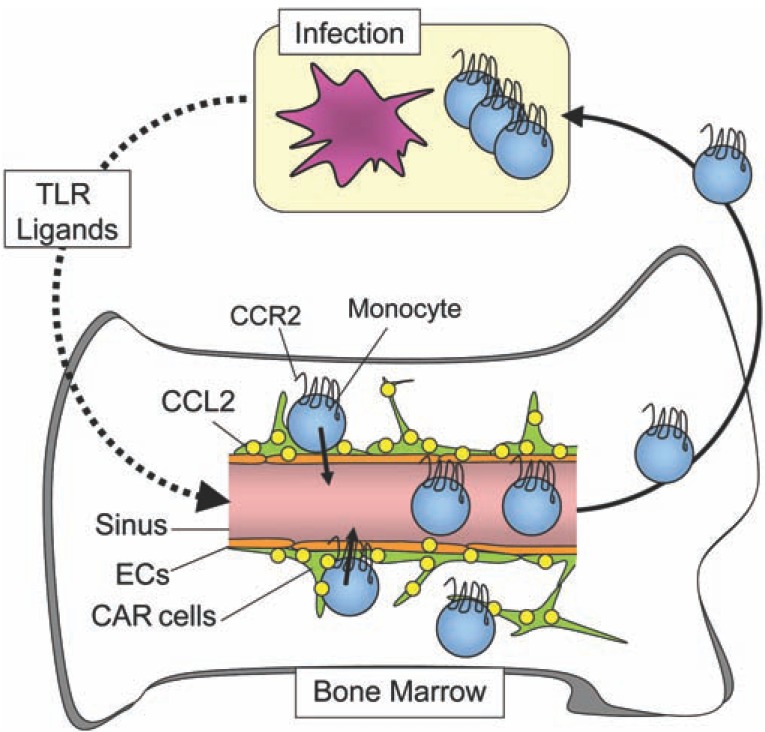
CAR cells respond to circulating microbial molecules and up-regulate the expression of another chemokine MCP1, which induces migration of inflammatory monocytes in the bone marrow cavity to sinusoidal endothelium to enter the circulation. In addition, some MCP1-expressing CAR cells located in intersinusoidal spaces might retain some monocytes in the bone marrow during infection.

Hematopoietic niches are thought to control hematopoiesis not only during homeostasis but also in inflammation. Recruitment of inflammatory monocytes as well as neutrophils from bone marrow to sites of infection is critical for innate immune defense against microbial pathogens. It has been shown previously that emigration of inflammatory monocytes from bone marrow into the circulation during infection with *Listeria monocytogenes*, an intracellular bacterial pathogen requires the chemokine receptor CCR2 [37]. The recent study has shown that the majority of CCR2^+^ inflammatory monocytes in the bone marrow cavities migrate to vascular sinuses and emigrate from the marrow into the circulation following low concentrations of the Toll-like receptor (TLR) ligands LPS [38]. In transgenic mice, in which cells expressing the chemokine CCL2, also known as monocyte chemoattractant protein-1 (MCP1), a ligand of CCR2, were marked using a bacterial artificial chromosome (BAC) mediated transgenesis with a linked GFP, the MCP1-GFP expression was significantly increased in CD45^-^Ter119^-^CD31^-^Sca1^-^PDGFRβ^+^ cells after administration of low concentrations of LPS and after infection with *Listeria monocytogenes*. Sorted MCP1-expressing cells expressed a high amount of CXCL12 mRNA following LPS administration. These results strongly suggest that the majority of MCP1-expressing cells following LPS administration are CAR cells [16, 38, 39]. In MCP1 conditional deficient mice, in which the MCP1 gene was deleted in MSCs and their progeny, including CAR cells and osteoblasts, the numbers of circulating Ly6C^hi^ inflammatory monocytes but not neutrophils was significantly reduced after administration of low concentrations of LPS [38, 39]. Thus, it is likely that MCP1 up-regulated in MSCs, CAR cells and/or osteoblasts is required for inflammatory monocyte emigration from the bone marrow into the circulation in response to low dose LPS. Together, CAR cells responded to circulating microbial molecules and an up-regulated expression of another chemokine MCP1, which induces migration of inflammatory monocytes in the bone marrow cavity to sinusoidal endothelium to enter the circulation (Fig. **2**). In addition, the results that some MCP1-expressing CAR cells were located in intersinusoidal spaces following the administration of low dose LPS are consistent with the fact that not all inflammatory monocytes exit the marrow and raise the possibility that MCP1-expressing intersinusoidal CAR cells retain some monocytes in the bone marrow during infection [38, 39] (Fig. **2**).

## SCF-EXPRESSING CELLS

Recently, mice containing the GFP gene knocked into the SCF locus (SCF-GFP mice), in which GFP was mainly expressed in cells surrounding sinusoidal endothelial cells, have been generated [17]. SCF-GFP expressing cells express CXCL12 mRNA at particularly high levels, and exhibit a homogeneous expression of PDGFRα and PDGFRβ [17]. Considering that CAR cells are the major producer of CXCL12 and SCF in the marrow, and exhibit a homogeneous expression of PDGFRα and PDGFRβ [16], SCF-GFP expressing cells would equate to CAR cells. In contrast, the results that SCF-GFP expressing cells do not express nestin mRNA and that in nestin-Cre, loxP- yellow fluorescent protein (YFP) mice EYFP-expressing cells were not detected along vascular sinuses suggest that SCF-GFP expressing cells are distinct from nestin-expressing cells [17]. As for their functional relevance, the numbers of HSCs were reduced when SCF was depleted from SCF-GFP expressing cells, using leptin receptor (Lepr) –Cre mice, based on the fact that mRNA expression of Lepr was highly restricted to SCF-expressing cells [17].

## THE VASCULAR NICHE: ENDOTHELIAL CELLS

The arteries enter the bone marrow through the cortical bone [40] and continue into typical capillaries that are confluent with a complex system of thin-walled sinusoids and which ramify throughout the marrow cavity. Some of these capillaries have an open lumen, and permits a slow blood flow and the passage of blood cells that are generated in the marrow. Highly purified HSCs can be identified as CD150^+^CD48^-^CD41^-^Lin^-^ cells and imaged in tissue sections [41]. In the bone marrow sections, many CD150^+^CD48^-^CD41^-^Lin^-^ HSCs are associated with sinusoidal endothelium, suggesting that endothelial cells function as a niche for HSCs (vascular niches) [41] (Fig. **1**). After myeloablation, selective activation of Akt1 in the endothelial cells of adult mice increases the number of HSCs and accelerates hematopoietic recovery [42]. These results do not rule out the possibility that endothelial cells indirectly enhance proliferation of HSCs by regulating other candidate niches closely adjacent to endothelial cells, including CAR cells and nestin-expressing cells; however, a recent study revealed that the numbers of HSCs were reduced when SCF was depleted from endothelial cells, using Tie2–Cre mice, indicating that endothelial cells are essential for maintenance of a pool of HSCs [17]. Of note, since expression of SCF was much lower in bone marrow endothelial cells compared with CAR cells (SCF-GFP expressing cells) [16, 17], there is the possibility that SCF produced by endothelial cells outside bone marrow might be involved in HSC maintenance. It will be important to identify additional requisite factors that are predominantly expressed in endothelial cells in the bone marrow and act directly on HSCs.

## CONCLUDING REMARKS

Although HSCs have been reported to be in contact with bone-lining osteoblasts and osteoblasts act as niches for HSCs, recent studies suggest that only a small subpopulation of HSCs reside in the endosteal niche. In contrast, *in vivo *roles for some mesenchymal cells in bone marrow cavities as HSC niches have been recently reported [16, 34]. Selective short-term ablation of nestin-expressing cells or CAR cells has suggested that nestin-expressing cells play an essential role in the maintenance of HSC numbers in part by retaining HSCs within bone marrow [34] and that CAR cells play a critical role in the maintenance of HSCs in a proliferative and undifferentiated state [16] although the role of these cellular niches in control of HSC quiescence remains unclear. Of note, it has been reported that CAR cells and nestin-expressing cells have the ability to differentiate into osteoblasts and adipocytes, raising the question if these cells are involved in bone formation and remodeling. The results that cells expressing EYFP from Lepr-Cre, loxP-EYFP mice were not detected along bone surface [17] suggesting that lepr-expressing cells, which might largely equate to CAR cells, do not give rise to osteoblasts during normal development. This is consistent with the fact that adult bone lining osteoblasts are derived from osterix-expressing precursors in the embryonic perichondrium of developing bone during homeostasis [43].

Of the candidate cellular niches for HSCs, SNO and Nes-GFP^+^, cells seem to be relatively rare non-hematopoietic cells that function as niches unique for HSCs. In contrast, CAR cells are relatively abundant and might function as a niche for both HSCs and more differentiated progenitors, including immune cells. In addition, CAR cells have long processes and form the reticular network within the bone marrow cavity. Considering that osteocytes, the most abundant cells in bone, communicate with each other through their processes, this raises the possibility that processes of CAR cells play a vital role in spatial and temporal regulation of their niche function in the bone marrow cavities. In this context, it will be important to address in future work what determines the specificity of niche-hematopoietic cell interaction, and to identify molecular mechanisms by which hematopoietic niches regulate maintenance, production and exit of various types of immune cells during homeostasis, microbial infection and inflammatory and myeloproliferative diseases. Furthermore, in addition to known PTH and its receptor PPR [8], and LPS receptor TLRs [38], molecules which act on cellular niches to induce or inhibit expression and/or secretion of requisite factors for hematopoietic cell behavior will be a pharmacological target for altering niche functions and this knowledge is likely to instruct the development of novel therapies in the future.
